# Management of post-acute COVID-19 patients in geriatric rehabilitation: EuGMS guidance

**DOI:** 10.1007/s41999-021-00575-4

**Published:** 2021-11-20

**Authors:** Jolanda C. M. van Haastregt, Irma H. J. Everink, Jos M. G. A. Schols, Stefan Grund, Adam L. Gordon, Else P. Poot, Finbarr C. Martin, Desmond O’Neill, Mirko Petrovic, Stefan Bachmann, Romke van Balen, Leonoor van Dam van Isselt, Frances Dockery, Marije S. Holstege, Francesco Landi, Laura M. Pérez, Esther Roquer, Martin Smalbrugge, Wilco P. Achterberg

**Affiliations:** 1 Department of Health Services Research and Care and Public Health Research Institute (CAPHRI), Maastricht University, P.O. Box 616, 6200 MD Maastricht, The Netherlands; 2 Department of Family Medicine, Maastricht University, Maastricht, The Netherlands; 3 Center for Geriatric Medicine, Agaplesion Bethanien Hospital Heidelberg, Geriatric Center at the Heidelberg University, Heidelberg, Germany; 4 School of Medicine, University of Nottingham, Derby, UK; 5 Verenso Dutch Association of Elderly Care Physicians, Utrecht, The Netherlands; 6 Population Health Sciences, Faculty of Life Sciences and Medicine, King’s College London, London, UK; 7 Trinity College Dublin Centre for Health Sciences, Tallaght University Hospital, Dublin, Ireland; 8 Section of Geriatrics, Department of Internal Medicine and Paediatrics, Ghent University, Ghent, Belgium; 9 Department of Rheumatology and Internal Medicine, Kliniken Valens, Valens, Switzerland; 10 Department of Geriatrics, Inselspital, Bern University Hospital, University of Bern, Bern, Switzerland; 11 Department of Public Health and Primary Care, Leiden University Medical Centre, Leiden, The Netherlands; 12 Leiden University Medical Center, Leiden, The Netherlands; 13 Department of Geriatrics and Stroke Medicine, Beaumont Hospital, Dublin, Ireland; 14 Department of Research GRZPLUS, Omring and Zorgcirkel, Hoorn, The Netherlands; 15 Geriatric Internal Medicine Department, Fondazione Policlinico Universitario A. Gemelli IRCSS, Rome, Italy; 16 Clinical Head of Outpatient Clinic and Geriatric Home Care, Intermediate Care Hospital Parc Sanitari Pere Virgili, Barcelona, Spain; 17 Research Group on Aging, Frailty and Transitions in Barcelona (RE-FiT BCN), Vall d’Hebrón Institut de Recerca, Barcelona, Spain; 18 Geriatric Service, University Hospital Sant Joan de Reus, Reus, Spain; 19 Department of Medicine for Older People, Amsterdam Public Health Research Institute, Amsterdam UMC, Vrije Universiteit Amsterdam, Amsterdam, The Netherlands; 20 Department of Public Health and Primary Care, Leiden University Medical Center, Leiden, The Netherlands; 21 Chair of the Guidance Committee Post COVID-19 Geriatric Rehabilitation, Verenso Dutch Association of Elderly Care Physicians, Utrecht, The Netherlands

**Keywords:** COVID-19, Guidance, Geriatric rehabilitation, Post-acute care, Quality assurance

## Abstract

**Purpose:**

To describe a guidance on the management of post-acute COVID 19 patients in geriatric rehabilitation.

**Methods:**

The guidance is based on guidelines for post-acute COVID-19 geriatric rehabilitation developed in the Netherlands, updated with recent insights from literature, related guidance from other countries and disciplines, and combined with experiences from experts in countries participating in the Geriatric Rehabilitation Special Interest Group of the European Geriatric Medicine Society.

**Results:**

This guidance for post-acute COVID-19 rehabilitation is divided into a section addressing general recommendations for geriatric rehabilitation and a section addressing specific processes and procedures. The Sect. “General recommendations for geriatric rehabilitation” addresses: (1) general requirements for post-acute COVID-19 rehabilitation and (2) critical aspects for quality assurance during COVID-19 pandemic. The Sect. “Specific processes and procedures”, addresses the following topics: (1) patient selection; (2) admission; (3) treatment; (4) discharge; and (5) follow-up and monitoring.

**Conclusion:**

Providing tailored geriatric rehabilitation treatment to post-acute COVID-19 patients is a challenge for which the guidance is designed to provide support. There is a strong need for additional evidence on COVID-19 geriatric rehabilitation including developing an understanding of risk profiles of older patients living with frailty to develop individualised treatment regimes. The present guidance will be regularly updated based on additional evidence from practice and research.

**Supplementary Information:**

The online version contains supplementary material available at 10.1007/s41999-021-00575-4.

## Key summary points

**Aim:**

To describe a guidance on the management of post-acute COVID 19 patients in geriatric rehabilitation.

**Findings:**

This guidance addresses general requirements for post-acute COVID-19 geriatric rehabilitation and critical aspects for quality assurance during the COVID-19 pandemic. Furthermore, the guidance describes relevant care processes and procedures divided in five topics: patient selection; admission; treatment; discharge; and follow-up and monitoring.

**Message:**

This guidance is designed to provide support to care professionals involved in the geriatric rehabilitation treatment of post-acute COVID-19 patients.

**Supplementary Information:**

The online version contains supplementary material available at 10.1007/s41999-021-00575-4.

## Background

The Severe Acute Respiratory Syndrome Coronavirus 2 (SARS-CoV-2) and its attendant syndrome, COVID-19, [1] is causing high morbidity and mortality rates around the world. Older people with frailty and underlying long-term conditions are highly susceptible to the disease [2], are more likely to develop severe symptoms and to have poor outcomes. COVID-19 mortality is highest in this group [3].

Much COVID-19 research has focussed on the acute phase, including symptomatic treatment, infection control and more recently drugs that modify the infection and attendant inflammatory response [4]. However, COVID-19 is a complex condition, with numerous complications including Acute Respiratory Distress Syndrome (ARDS), sepsis, kidney failure, heart failure, coagulopathy with thromboembolism and multi-organ failure. These conditions each contribute to disability which can persist past the acute phase. In addition, a number of other problems are evident post COVID-19, including critical illness polyneuropathy (CIP), critical illness myopathy (CIM), post-intensive care syndrome (PICS), severe deconditioning, acute sarcopenia, post-traumatic stress disorder (PTSD) and various other somatic, cognitive, emotional and psychosocial problems, which may result in prolonged functional decline and reduced quality of life [4–13]. Carfi and colleagues assessed the persistent symptoms in patients who were discharged from hospital after recovery from COVID-19 and concluded that (on average) 2 months after disease onset, 87% of these patients reported persistence of at least one symptom. More than 50% experienced persistent fatigue, 43% suffered from dyspnoea and 27% experienced joint pain. The rate of these symptoms is higher among older people [14, 15]. Intensive medical input and adapted physical activity may allow post-acute COVID-19 patients to resume their pre-infection lives and habits sooner [15]. According to the “Gemelli Against COVID-19 Post-Acute Care Study Group” the multi-organ involvement of COVID-19 requires an interdisciplinary approach in which the geriatrician or physician specialised in care for older people, may serve as a case manager [16]. Persistent symptoms are not restricted to those hospitalised with COVID-19 and many people who had initially mild disease report an array of long-term sequelae, with persistent fatigue similar to post-viral fatigue syndrome being among the most widely reported [17].

Many COVID-19 patients require considerable time to return to their previous life pattern, and many have complex rehabilitation needs. This makes multidisciplinary rehabilitation an essential part of COVID-19 recovery [15, 18, 19]. Within the group of COVID-19 patients with complex rehabilitation needs, a subgroup of vulnerable older patients can be distinguished. This subgroup primarily consists of frail, multimorbid older people who have several premorbid long-term conditions and were often already (partly) dependent on formal and/or informal care before the onset of COVID-19. However, it also includes older people who were relatively healthy before but who experienced severe functional decline and deterioration in activities of daily living alongside other phenotypic characteristics of frailty following COVID-19. These older patients present a complex mix of new and pre-existing disability, their response to rehabilitation may be attenuated by frailty and cognitive impairment, and their ability to participate may be limited by environmental factors including social isolation and care dependency. A different, more comprehensive, approach to rehabilitation is required; geriatric rehabilitation.

Geriatric rehabilitation is defined by The Geriatric Rehabilitation Special Interest Group of the European Geriatric Medicine Society (EuGMS), as a multidimensional approach of diagnostic and therapeutic interventions, the purpose of which is to optimise functional capacity, promote activity and preserve functional reserve and social participation in older people with disabling impairments [20]. People eligible for geriatric rehabilitation are often affected by multimorbidity and geriatric syndromes [20]. They need care from a multidisciplinary team specialised in rehabilitation of older people.

Geriatric rehabilitation can be offered in an inpatient or outpatient setting. Grund and colleagues [21] identified major differences between EuGMS member countries with regard to the availability of geriatric rehabilitation in general and how it is structured and delivered. Inpatient geriatric rehabilitation is provided in nursing homes, geriatric rehabilitation centers, geriatric rehabilitation hospitals, and acute care hospitals. Approximately two thirds (*n* = 20) of the EuGMS member countries formally recognise geriatric rehabilitation as a separate discipline. In the majority of these countries the multidisciplinary geriatric rehabilitation teams are led by a geriatrician or physician specialised in geriatric rehabilitation and include nurses, physiotherapists, occupational therapists, speech therapists, social workers and dieticians. In approximately a quarter of the member countries with geriatric rehabilitation, a psychologist is also part of the multidisciplinary team [21]. Furthermore, in member countries where geriatric rehabilitation is not recognised as a separate discipline and specialised geriatric rehabilitation facilities do not exist, geriatric rehabilitation is often provided in other care facilities such as care homes or outpatient facilities. It often starts in acute hospitals prior to discharge.

Before the COVID-19 pandemic, several studies into the effects of geriatric rehabilitation have shown promising results, including improved function [22–24], increased quality of life [25], and reduced rates of institutionalisation and mortality [22]. Facilities providing geriatric rehabilitation have a crucial role in enabling older community-living adults to return to their previous living situation and resume their lifestyle, either through recovery of function or the use of adaptations. However, in many countries there has been an inadequate provision of rehabilitation services for older people to date, arising from a lack of policy framework for geriatric rehabilitation and a lack of available geriatric medicine resources. This has been, to an extent, because of a focus on the cost of rehabilitation without a view to cost-effectiveness that adequately captures the impact of rehabilitation on patients’ well-being [26].

The need for adequate and specialised geriatric rehabilitation services has increased during the COVID-19 pandemic. Due to the very heterogeneous presentation of COVID-19 in combination with age-related problems such as frailty, cognitive impairments and multimorbidity, the treatment of older post-acute COVID-19 patients is very challenging. However, rather than responding to the pandemic through augmentation of capacity in geriatric rehabilitation (as might have been expected given the adverse effect on older patient cohorts) many health systems have used rehabilitation facilities and teams to shore up acute services. Availability of rehabilitation resource has diminished rather than improved during the pandemic [27, 28]. Meanwhile, recovery post-COVID-19 is sufficiently different from normal rehabilitation scenarios to mandate national guidelines whilst, at the same time, those national guidelines have often made only scant or passing guidance to older people [29]. Recently several systematic reviews have been conducted on COVID-19 rehabilitation and Barker-Davies and colleagues introduced a consensus statement for post COVID-19 rehabilitation [4, 30, 31]. However, a guidance specifically focussed on the management of vulnerable older (post-acute) COVID-19 patients in *geriatric* rehabilitation is currently lacking.

Against this background, there is a strong need for consensus and/or evidence-based guidance for the management of post-acute COVID-19 patients in geriatric rehabilitation. The Geriatric Rehabilitation Special Interest Group of the EuGMS developed this guidance for the management of post-acute COVID-19 patients in geriatric rehabilitation. In this guidance, “post-acute COVID-19” refers to patients who have survived the acute phase of the disease at home or in a hospital, and are currently recovering. The guidance was based on guidelines for post-COVID-19 geriatric rehabilitation developed in the Netherlands [32, 33], updated with recent insights from literature, related guidance from other countries and disciplines, and combined with experiences from experts in countries participating in the Geriatric Rehabilitation Special Interest Group of the EuGMS.

This article describes this guidance. It is proposed that the guidance will be a living document, updated on a regular basis based on additional evidence from practice and research including the results from the EU-COGER study, which explores the effects of geriatric rehabilitation on (post-acute) COVID-19 patients [34]. This guidance can not only be used in geriatric rehabilitation facilities, but also in facilities providing care to similar patients in countries where geriatric rehabilitation is not formally recognised. The recommendations in this guidance are formulated in a relatively general way to make them applicable in all member countries. If necessary, on a national level the recommendations can be further specified to tailor them to the local circumstances.

## Methods

Guidance developed by Verenso, the Dutch Association of Elderly Care Physicians and Social Geriatricians, was used as a starting point. The Dutch guidance for COVID-19 geriatric rehabilitation consisting of an inpatient [32] and outpatient module [33], was developed by 32 experts in the field of geriatric rehabilitation, including researchers, physicians specialised in geriatric rehabilitation and consultants in rehabilitation. They based their recommendations on scientific literature, available evidence from practice, and guidance in the treatment of chronic obstructive pulmonary disease (COPD) and Post-ICU COVID-19 rehabilitation [35–37]. In addition, the results of the EuGMS Geriatric Rehabilitation Special Interest Group survey on best practice in geriatric rehabilitation in Europe, which also analysed the changes due to the COVID-19 pandemic, were taken into account [38]. As a first step, the main aspects of this guidance were translated by IE en JvH into an English version and reviewed by four geriatric rehabilitation experts to consider whether they needed augmentation or adaptation for application outside the Dutch context (authors AG, JS, SG and WA) of the Geriatric Rehabilitation Special Interest Group of the EuGMS. They were asked to add relevant and recent insights from member countries to make the guidance applicable for the European context. As a second step, a concept version of the guidance was developed and sent for review to the other members of the Geriatric Rehabilitation Special Interest Group of the EuGMS, the board of the EuGMS, and the coordinators of the EU-COGER study [31]. They were asked to assess if the guidance would be applicable in their country and whether they wanted to add, delete or adapt aspects of the guidance based on recent literature and/or their expert opinion. Based on their responses (*n* = 13) a final version of the guidance was developed, which is described in the current paper. The guidance is as evidence-based as was possible given the maturity of the scientific literature on COVID-19 rehabilitation at the time of writing. We have taken a pragmatic approach, drawing on evidence where available and using expert consensus based upon clinical experience to complete the guidance.

## Recommendations

This guidance for post-acute COVID-19 rehabilitation is divided into a section addressing general recommendations for geriatric rehabilitation and a section addressing specific processes and procedures. The sect. “General recommendations for geriatric rehabilitation” addresses: (1) general requirements for post-acute COVID-19 rehabilitation and (2) critical aspects for quality assurance during COVID-19 pandemic. The Sect. “Specific processes and procedures”, addresses the following topics: (1) patient selection; (2) admission; (3) treatment; (4) discharge; and (5) follow-up and monitoring.

### General recommendations for geriatric rehabilitation

1. *General requirements for post-acute COVID-19 rehabilitation*.
  Providing post-acute COVID-19 geriatric rehabilitation that is safe both for the patient and the care provider requires additional arrangements and equipment, which is not routinely required for other conditions routinely managed in geriatric rehabilitation. First, it is essential to have high quality personal protective equipment (PPE), following the current (national) guidelines and requirements. In addition, staff, patients, informal caregivers and other visitors should be informed on how to use which PPE and when. Furthermore, management guidelines on the time frame patients discharged from hospital are still contagious should be available and known by staff, patients and their informal caregivers in the geriatric rehabilitation facility. COVID-19 test facilities should be available for staff, patients, informal caregivers, volunteers and other visitors.
  Other equipment which is not necessarily routinely available in geriatric rehabilitation facilities but necessary for safe care after COVID-19 is: (1) oxygen concentrators for patients still in need of extra oxygen; (2) medical oxygen cylinders or central oxygen lines for patients still in need of high flow oxygen; (3) pulse oximeters; and (4) a tracheostomy suction pump. Optional equipment which can be helpful during rehabilitation of (post) COVID-19 patients is: a Threshold Inspiratory Muscle Trainer to exercise inspiratory muscle strength, and a breathe (resistance) trainer. Furthermore, attention should be paid to training the staff in using the services and materials mentioned and in monitoring the patients in a proper and safe way.
  How care is organised within institutions requires some attention if rehabilitation is to be effective. Ideally, patients with COVID-19 admitted to (inpatient or outpatient) geriatric rehabilitation should not be contagious anymore, because a recent study [38] showed that infection prevention and control procedures for COVID-19 patients could limit treatment opportunities. Rehabilitation within a small single room can be difficult. Careful attention to cohorting of staff and patients can, though, be used to enable whole areas of buildings to be identified as “red” (COVID positive) or “green” (COVID negative), which has potential to open up corridors, gyms and living areas to support rehabilitation [39]. The movement of staff between red and green settings must be carefully considered and minimised, with appropriate decontamination facilities (soap and water, and space for donning, doffing and disposing of Personal Protective Equipment) between zones. This also requires consideration in people’s own homes, where staff movement between COVID-19 positive and negative patients should be minimised, particularly because of the limited resource for effective decontamination between home visits.
2. *Critical aspects for quality assurance during COVID-19 pandemic*.
  Grund and colleagues performed an online survey to assess pandemic-related changes on structure and process level in geriatric rehabilitation facilities of various European countries [38]. They found that various processes and procedures were negatively affected by the pandemic, including comprehensive geriatric assessment, regular team meetings, daily rounds, caregiver training, shared decision making with patients and their relatives, transitional care, structured discharge planning, outpatient geriatric rehabilitation after inpatient geriatric rehabilitation, and outcome measurement after discharge [38]. Many of these negative effects were due to necessary hygiene requirements like distancing, contact restrictions and handling of protective equipment. These pandemic-related changes may have a significant impact on the quality of care and outcome in geriatric rehabilitation of post-acute COVID-19 patients. It is important for multidisciplinary teams to be aware of these potential threats to quality of care, to ensure that process and outcome measures are in place to capture their impact on patient care and that measures are taken to mitigate against this.
  Testing for COVID-19 raises particular issues for how care is organised. Reverse Transcriptase-Polymerase Chain Reaction (RT-PCR) tests are widely regarded to be the gold-standard for diagnosis of COVID-19 but can remain positive for a substantial period after initial infection, even once infectivity has passed [45]. RT-PCR testing for SARS-CoV-2 should therefore not be routinely conducted at admission or for surveillance in rehabilitation facilities where patients have recently tested positive for COVID-19 [40]. Where patients have persistent or recurrent symptoms compatible with COVID-19, an RT-PCR test must be conducted and a positive result should be treated as likely COVID-19 infection. Lateral flow and automated antigen tests for SARS-COV-2 are less sensitive and specific than RT-PCR but tend to be positive only during the period of maximum infectivity [41]. Regular surveillance for staff can be conducted using either RT-PCR or antigen testing but test frequency must be adjusted to take account of test sensitivity [42]. There is limited evidence that regular surveillance for patients adds much over and above routine screening of staff and standard IPC measures and it is not recommended. However, the current regulations and laws of the respective country should be observed for measures to protect against infection.

### *Specific processes and procedures*

Below, the following phases in geriatric rehabilitation are described: (1) patient selection; (2) admission; (3) treatment; (4) discharge; and (5) follow-up and monitoring. Due to the heterogeneity of the target population of post-acute COVID-19 patients in geriatric rehabilitation, the recommendations made in this section cannot directly be related to specific patient profiles. The recommendations can be used as a checklist based on which care professionals can decide which recommendations to apply tailored to the specific care needs of their patients.

1. *Patient selection phase*.
  With regard to selecting patients for post-acute COVID-19 rehabilitation in general, Wade indicates there are no specific symptoms or signs that enable prediction of the impact of COVID-19 on long-term functional status and hence the need for rehabilitation after the acute phase [19]. Identifying which patients are likely to respond to geriatric rehabilitation is notoriously difficult and there is a substantial risk of subjective bias in judging outcomes. Therefore, an inclusive approach should be adopted to inviting patients for rehabilitation [43]. If a problem or concern is identified in a patient which will not obviously resolve without intervention soon, then the patient should be referred to multidisciplinary rehabilitation [19]. Younger patients who have multiple long-term conditions and/or frailty may also be admitted to geriatric rehabilitation when their level of functioning is comparable to older patients who would be likely to benefit.
  Several areas should be considered when reviewing the situation of a person with COVID-19: any new problems or symptoms that have occurred as a consequence of the COVID-19 infection; the ability to undertake (ADL and IADL) actions the person wishes to do at the level they expect; experiencing (new or more severe) physical or psychological problems; and any other problems in daily life that concern the patient [19]. Furthermore, COVID-19 may be associated with decline in cognitive functioning [44, 45]. Therefore, special attention should be paid to the assessment of cognitive impairments. Cognitive impairments might reduce the trainability of patients, which could negatively influence the rehabilitation potential of these patients.
  Besides these special points of attention, the selection procedure of COVID-19 patients is comparable to the procedure recommended for other patient groups. This procedure consists of a geriatric needs assessment to gain more insight in whether and how an older patient may benefit from rehabilitation. This assessment is preferably conducted by a geriatrician or a physician specialised in geriatric rehabilitation and/or delegates from the wider rehabilitation MDT (if necessary in consultation with other disciplines such as a consultant in rehabilitation, lung specialist, internist, etc.). Based on geriatric needs assessment the frailty status, functional prognosis, trainability, cognition and motivation of the patient can be assessed, and it can be decided if a (post-acute) COVID-19 patient needs additional treatment and whether this treatment should be monodisciplinary care or multidisciplinary rehabilitation. Furthermore, the geriatric assessment should conclude whether the rehabilitation should be provided in a geriatric rehabilitation facility, medical specialist rehabilitation facility or specialised pulmonary rehabilitation facility (if available), and the choice should be tailored by matching the patients’ needs to the medical, therapist, and nursing provision available in the receiving facility.
  Lastly, based on the geriatric needs assessment, a decision for inpatient care, outpatient care or a combination of the two should be taken. Outpatient geriatric rehabilitation can be provided at an outpatient clinic or in the home of the patient. The decision for inpatient or outpatient care, also depends on the available services in the member countries, local circumstances, and the availability of informal care for the patient. All may be possible within the context of COVID-19 but consideration of the risks of infection between patients, visiting health professionals and (potentially frail) cohabitees must be considered alongside which treatments can be offered at each venue.
  Within the target population of post-acute COVID-19 geriatric rehabilitation described above, two categories of patients can be distinguished based on their previous treatment setting: COVID-19 patients discharged from hospital and patients who managed COVID-19 at home or in a residential care setting. Although a large majority of patients are admitted to rehabilitation after hospitalisation, a small minority come from home or a residential care setting. The size of this group differs between European countries and is related to the nature of the health care system and regulations in different countries. The two categories are described below:
  *Post-acute COVID-19 patients admitted after hospitalization*.
  Patients can be admitted to geriatric rehabilitation after discharge from hospital either following an ICU admission or ward-based care. COVID-19 patients discharged from hospital after ICU admission can be affected by a variety of problems, such as the post-intensive care syndrome (PICS), including impairments in physical, cognitive, and/or mental functioning [46–48]. These patients are often severely deconditioned, oxygen-dependent, have limited pulmonary and respiratory reserve, and may have comorbidities which have decompensated during their acute admission. Furthermore, they are often experience psychological problems [such as post-traumatic stress disorder (PTSD)] and cognitive problems. They require extra attention towards malnutrition, breathing issues, swallowing impairment and oropharyngeal dysphagia. A focus on prevention of sputum retention, aspiration and pressure ulceration is required.
  Within the group of COVID-19 patients discharged from hospital without ICU admission, two subgroups can be distinguished. The first subgroup consists of patients with severe sarcopenia, who are severely deconditioned, combined with pre-existing frailty and complex health problems. Initially, these patients are often dependent on additional oxygen and have moderate psychological and cognitive problems.
  The second subgroup consists of older patients who were not substantially frail before COVID-19 infection, but who need an extended period of recovery. This recovery is mainly aimed at becoming independent from oxygen, regaining mobility and improving physical condition. They may be comorbid to some extent, but are not severely impaired by these comorbidities. In general they only have minor or no psychological or cognitive problems.
  *Post-acute COVID-19 patients admitted from home*.
  Within the group of COVID-19 patients admitted to geriatric rehabilitation from home, two subgroups can be distinguished. The first group are COVID-19 patients who are independently living or living in a residential care setting. Due to COVID-19 they have a relatively high care dependency, which makes the care burden for their caregivers too high. Hospital admission is considered not necessary or appropriate for this group because they do not need additional oxygen, or oxygen is already being administered at home. Patients in this group are often very frail and have a high risk of impaired functional outcome and mortality [49]. Their clinical trajectory is hard to predict. Some of these patients will recover, but they may need a relatively long period of recovery and/or rehabilitation; others will not be able to continue living independently (especially in case of a lack of informal support) and are admitted to a residential or long term care facility.
  The second subgroup are older patients who were not substantially frail before COVID-19 and who have initially been discharged home after hospitalization, but who need rehabilitation because they experience substantial problems with their activities of daily living due to COVID-19. This rehabilitation is mainly aimed at improving physical condition and the ability to perform activities of daily living.
2. *Admission phase*.
  In Table 1 the main activities during the admission phase are summarised. These are divided into activities generally performed in geriatric rehabilitation treatment, and activities specifically related to COVID-19 problems.
3. *Treatment phase.*
  In this section three aspects of the treatment phase are descibed: general treatment goals, treatment goals per domain, and tailoring treatment program to changing capacity and needs of patients

- *General treatment goals* The general treatment goal of post-acute COVID-19 geriatric rehabilitation is to improve the complete health and/or functional status of older and/or frail patients who have had COVID-19 infection, and to prevent and treat the physical, functional and psychological impairments resulting from this infection. Post-acute COVID-19 geriatric rehabilitation is aimed at having patients return to their original living situation where they are able to perform activities and participate in society, at the same level of pre-COVID-19 times.
- *Treatment goals per domain* Besides the overall rehabilitation goals, more specific treatment goals and related actions are distinguished per domain in Table 2 (the somatic, functional, psychological, existential and social domain). Part of these cover geriatric rehabilitation patients in general, and part is specifically focussed on post-acute COVID-19 patients. Furthermore, in Appendix 1 the goals for each discipline included in the multidisciplinary geriatric rehabilitation teams are described for the physician, nurse, physiotherapist, occupational therapist, dietician, speech and language therapist, psychologist, and social worker. The disciplines involved in the treatment of COVID-19 patients may differ per patient based on the specific symptoms and comorbidities of the patient. For care facilities with fewer disciplines in the multidisciplinary teams, certain goals may be assigned to other disciplines. Collaboration with disciplines from other care organisations should be organised, if needed to meet the needs of the patients. Within geriatric rehabilitation the interchange between disciplines is very important and the care professionals involved in geriatric rehabilitation should have a flexible and interdisciplinary work approach. Furthermore, it is recommended that the geriatric rehabilitation team collaborates closely with a (clinical) pharmacist given the impact of polypharmacy on rehabilitation and recovery and the changes to drug regimens which are commonly a part of COVID-19 care.
- *Tailoring treatment programme to changing capacity and needs of patients*
  The group of post COVID-19 patients who can benefit from geriatric rehabilitation is very heterogeneous with regard to endurance capacity and course of recovery. Clinical experiences from the pandemic have shown that some patients who had a long ICU admission or who are very old, have a very low endurance capacity at admission to geriatric rehabilitation but recover relatively quickly. Other patients have a slower speed of recovery and a low endurance capacity. Currently, there is still a lack of insight in factors predicting the course of recovery in frail older post COVID-19 patients. Therefore, it is important to individualise rehabilitation treatment to take account of the observed differences in course of recovery. The multidisciplinary geriatric rehabilitation team should continuously assess which treatment intensity fits which patient at which moment. Furthermore, in countries where outpatient geriatric rehabilitation is available, the multidisciplinary geriatric rehabilitation team should also continuously assess in close collaboration with the patients and their informal caregivers, whether outpatient treatment is appropriate and safe for the patient and feasible for the informal caregivers. During ambulatory treatment, the use of eHealth should be considered for telerehabilitation, monitoring and consultation purposes.
  In Table 3 the main activities during treatment in geriatric rehabilitation are summarised.
  4. *Discharge phase*.
    Predicting the time of discharge at admission is difficult among COVID-19 patients due to the unpredictable course of the disease and recovery process. With regard to discharge planning, it is important that the patient and their family (if applicable) are actively involved in this phase, including discussion of the available options for (formal and informal) care and/or medical aids at home. The activities during discharge from geriatric rehabilitation are presented in Table 4. It is important to start discharge activities early during the rehabilitation process, because some activities will take some time (e.g. organising materials/adaptations in the home environment and organising follow-up care). Vaccination status should be revisited prior to discharge. It is increasingly likely that older people will have been vaccinated given the extensive coverage across the older age group—but those who have previously declined vaccination may choose to revisit this decision in light of having experienced COVID-19, as recurrent infection is a possibility after acute illness [50, 51]. In addition, as booster programmes become established internationally, ensuring that patients are up-to-date with COVID-19 vaccination will be important, as they may have missed a booster during hospital admission. Finally, the impact of the combined effects of influenza and COVID-19 on older people over the coming winter have been extensively modelled [52], influenza vaccination should be actively encouraged in older patients who have not yet received this.
  5. *Follow-up and monitoring*.
    In this section two aspects are descibed: follow-up after discharge and monitoring during all phases of geriatric rehabilitation.
    *Follow-up after discharge* After discharge patients should be followed up, because COVID-19 can have various long term somatic, cognitive, emotional and psychosocial consequences [4–13]. However, insight is lacking in the most appropriate timing and frequency of follow-up assessments among older, frail and multimorbid patients discharged from geriatric rehabilitation. The currently running EU-COGER study conducts follow-up measurements after 6 weeks and 6 months and assesses ADL functioning, perceived quality of life, and social participation at follow-up [34]. In addition to these measurements, we recommend to assess at follow-up the availability of informal caregivers, caregiver strain and additional measures if indicated based on the status of the patients at follow-up. Monitoring during follow-up can be performed by the general practitioner of the patient (or another member of the primary care team, such as a nurse practitioner). eHealth applications can be used for monitoring purposes. Preferably widely acknowledged measurement instruments should be used for this monitoring (see next section). In case of problems in these domains, the patients should be referred to other care professionals if indicated, such as a physiotherapist, occupational therapist, dietician, speech and language therapist, psychologist, social worker, specialised nurse, geriatrician or physician specialised in geriatric rehabilitation.
    *Monitoring during all phases of geriatric rehabilitation* During all phases of geriatric rehabilitation discussed in the previous sections (selection, admission, treatment, discharge, and follow-up) it is essential to screen and monitor the functioning and health status of the patients to detect deterioration and to act upon it. Table 5 presents a number of widely acknowledged measurement instruments which are recommended to be administered before, during and after geriatric rehabilitation. The measurements are mainly based on the measurements included in the ongoing EU-COGER study [34] and are categorised based on the domains proposed by the International Classification of Functioning, Disability and Health (ICF): body functions and structures, activities, participation, the physical and social environment and personal factors [53]. The final choice for instruments should depend on the instruments which are available and commonly used in the member countries and the status of the patient. However, preferably measurements from each ICF domain should be used to adequately monitor the functioning and health status of geriatric rehabilitation patients. Besides the measurements suggested in Table 5, it is recommended to regularly (for example on a weekly basis) administer rapid diagnostic tests, for example Lateral Flow Tests, during geriatric rehabilitation of post-acute COVID-19 patients, although the positive predictive validity and hence clinical utility of these will vary as background prevalence changes and such policies should be subject to regular review in conjunction with public health experts [54]. Furthermore, it is highly recommended to create an internationally agreed upon and standardised register for COVID-19 patients in geriatric rehabilitation, including a selection of widely used measurement instruments. This would facilitate comparison between care organisations and health care systems (countries), and could also be used for research on COVID-19 rehabilitation in general, and for developing a risk stratification for post COVID-19 patients specifically. This could help to further tailor the geriatric rehabilitation treatment to the risk profile of older patients affected by COVID-19 against a background of frailty and multimorbidity. Facilities providing geriatric rehabilitation to post-acute COVID-19 patients are invited and encouraged to participate in the EU-COGER study [34] or join an international standardised register for COVID-19 patients in geriatric rehabilitation, after the COGER study is completed.

**Table 1:** Main activities in geriatric rehabilitation during admission*

Activities during admission phase
*General*
Performing comprehensive geriatric assessment (CGA) to gain insight in the frailty status, functional prognosis, trainability, cognition and motivation of the patient (or use data from recently performed CGA)
Anamnesis: somatic, functional, psychological, existential and social domain
Performing measurements and screening on all domains (see also Sect. 5 Monitoring and follow-up)
Reporting on the findings of the CGA in the (electronic) patient file
Formulating treatment goals with the patient and his/her relatives, resulting in a treatment plan (by applying shared decision making)
Discussing policy with patient and his/her family (if applicable) in case of deterioration (advance care planning, non-COVID-19-related)
Starting the discharge-process as soon as possible
Discussing expected duration of rehabilitation treatment (expected week of discharge)
Discussing expected discharge location (home, residential care setting, nursing home)
*COVID-19*
Specific focus in all domains of CGA on COVID-19-related complications and symptoms
Due to contact restrictions/lockdowns it is important to make a structured caregiver communication plan, in which consultations with care professionals are planned in advance, to guide the informal caregivers through the rehabilitation process and to provide them the opportunity to ask questions (e.g. at admission, in-between (frequency depends on the expected length of stay), and prior to discharge). Understanding the shielding requirements of caregivers is important as many will be vulnerable to COVID-19
Discussing the relevant COVID-19 policies with patient and family (if applicable) with regard to testing, visiting, safety measures, quarantine, use of protective measures (PPI), vaccination and hygiene
Discussing policy with patient and their family (if applicable) in case of deterioration (advance care planning, COVID-19-related)

*Based on Verenso (2020) and supplemented and adapted by the EuGMS Geriatric Rehabilitation Special Interest Group

**Table 2:** Treatment goals and actions per domain*

*Somatic domain*
*General*
Improve the general condition of the body, stamina and muscle strength, and treatment of sarcopenia (including sufficient attention for the balance between what is possible and what is not possible given current impairments, and for nutrition and exercise)
Optimizing the somatic part of geriatric syndroms (e.g. incontinence—treatment or urinary tract infection; insomnia—day/night regulation, medication; impaired vision—contact caregiver (glasses); hearing loss—including treatment of cerumen impaction)
Improving nutritional status (by sufficient food intake and/or attention for increased protein, vitamin and energy intake)
*COVID-19*
Medication review and optimising medication use (including attention to anticholinergic burden for those with cognitive impairment)
Regulating and/or dismantling the use of oxygen
Managing cannula care or post-cannula problems, proper management of IV lines or PICC lines
Stabilise and/or treat comorbidity [e.g. hypertension, pain (peripheral neuropathic pain post ICU)]
Preventing functional loss due to complications (e.g. preventing contractures, prevent and treat pulmonary complications and pressure ulcers)
Managing and/or improving functional swallowing capacity (e.g. functional speech therapy and clinical nutrition)
*Functional domain*
*General*
Assisting patients to manage personal activities of daily living without the assistance of another person (i.e. improving self-management skills). If this is not possible, the goal is to minimise the need for external assistance through the use of adaptive techniques and equipment, aiming at the highest achievable level of independence [37]
Improving mobility: independent transfers, walking (with or without walking aids), climbing stairs
Assessing and adapting the home environment if necessary
*COVID-19*
Improving swallowing and breathing techniques, speech techniques, breathing power, and coughing techniques (in case of respiratory obstruction)
Gaining insight in reduced energy levels including learning compensation strategies
*Psychological domain*
*General*
Gaining insight in cognitive changes and learning strategies to compensate for this
*COVID-19*
Stabilising mood (timely diagnosis and treatment of depression, fear and PTSD; assessing possible impairing psychological factors)
Supporting patient and family (if applicable); timely identification of psychological complaints related to post-intensive care syndrome-family (PICS-F), and emotional overload
*Existential domain*
*General*
Mapping sources for resilience and supporting resilience
Exploring questions regarding identity, values and meaning of life
*COVID-19*
Support in processing experiences around suffering and fear of dying
Supporting to overcome COVID-19 as a life event
Spiritual counselling
*Social domain*
*General*
Improving social participation, based on personal goal setting
Mobilising practical and social support
Supporting informal caregivers and the patients’ social network
*COVID-19*
Reintegration into social life within COVID-19-related social restriction measures

*Based on Verenso (2020) and supplemented and adapted by the EuGMS Geriatric Rehabilitation Special Interest Group

**Table 3:** Main activities during geriatric rehabilitation treatment*

Activities during treatment phase
*General*
Execution of the treatment plan by the disciplines involved
Continuous monitoring of signs and symptoms and course of recovery (see Sect. 5 Monitoring and follow-up)
Evaluation and adaptation of treatment goals and treatment plan if necessary
Actively engaging patient and family/informal caregivers (if applicable) in the evaluation and treatment decisions, and support them in shared decision making if needed
Monitoring and reporting the progress in the (electronic) patient file
*COVID-19*
Specific focus on monitoring COVID-19-related symptoms, complications and their interaction with other comorbidities, next to regular functional outcome measurement
Specific focus on monitoring COVID-19 contagiousness
Check vaccination status/indication

*Based on Verenso (2020) and supplemented and adapted by the EuGMS Geriatric Rehabilitation Special Interest Group

**Table 4:** Main activities during discharge from geriatric rehabilitation*

Activities during discharge phase
*General*
Prepare the patient and their relatives/informal caregivers for discharge and support them in the transition of the patient to the home environment or other care setting
Performing measurements and screening on all relevant domains (see Sect. 5 Monitoring and follow-up)
Assess whether there is a need for medical aids (including oxygen) in the home
Assess whether there is a need for aftercare in the home (such as outpatient rehabilitation, primary care physiotherapy, home care, etc.)
Discuss the necessary preparations with the patient, family/informal caregivers (if applicable)
Prepare timely and detailed transfer of patient records to primary care professionals
*COVID-19*
Discussing relevant COVID-19 policies with patient and family (if applicable) with regard to safety measures
Specific focus in measurement and screening on COVID-19-related complications and their interaction with other comorbidities, next to regular functional outcome measurement
Assess the home environment and social network of the patient, bearing in mind current social distancing policies

*Based on Verenso (2020) and supplemented and adapted by the EuGMS Geriatric Rehabilitation Special Interest Group

**Table 5:** Measurements instruments for the five phases of post-acute COVID-19 geriatric rehabilitation*

Outcome	Instrument	Patient selection	Admisson^#^	Treatment	Discharge	Follow-up
*Body functions and structures*						
Frailty	CFS	X	X	O	O	O
Nutrition/malnutrition	BMI (kg/m^2^)/SNAQ65, MNA, MUST, NRS, Weight loss, or equal measurements	0	X	O	X	X
Comorbidity	wFCI	X	X	O	O	O
Cognition	USER, MOCA, MMSE, Demtect	X	X	O	X	O
Delirium	DOS, 4AT, SQiD or equal measurements	X	X	O	O	O
Depression	HADS, GDS-15	X	X	O	X	O
Pain	NRS-P	X	X	O	X	O
Skin	Pressure ulcer: yes/no	X	X	O	X	O
Symptoms	Fatigue VAS, Dyspnoea VAS, Saturation, Blood gas	X O	X O	O O	X O	O O
Mobility/balance	TUG, CST, SPPB	O	O	O	O	O
Muscle strength	MRC biceps/quadriceps	O	O	O	O	O
Post-traumatic stress syndrome	PTSS: yes/no	–	–	–	O	O
Speech	Speech problems: yes/no	O	O	O	O	O
Swallowing	MWST, Swallowing problems: yes/no	O	O	O	O	O
*Activities*						
Activities of daily living	BI, USER, FIM	X	X	X	X	X
*Participation*						
Quality of life	EQ-5D-5L	X	X	O	X	X
Social participation	FAI, USER-P	–	–	–	–	X
*Physical and social environment*						
Residency	Premorbid residency of participants	X	X	–	–	–
Destination	Discharge destination after GR	–	–	–	X	–
Informal care	Availability of informal caregivers	X	X	X	X	X
*Personal factors*						
Age	Year of birth/age	X	X	-	-	–
Gender	Male/female	X	X	–	–	–
COVID-19	SARS-CoV-2 diagnosis confirmed by PCR or Serology	X	X	–	–	–
Hospitalisation	Number of days in hospital before GR	X	X	–	–	–
Intensive care	Number of days at the ICU before GR	X	X	–	–	–
Complications before admission GR	Complications before admission to GR: thromboembolism, delirium, pressure ulcer	X	X	–	–	–
Complications during GR	Complications during inpatient GR: thromboembolism, delirium, pressure ulcer	–	–	–	X	–
Length of stay	Length of stay in inpatient GR	–	–	–	X	–
*Informal caregiver*						
Caregiver strain	CSI	–	–	X	X	X

*Based on Grund (2021) and Verenso (2020) and supplemented and adapted by the EuGMS Geriatric Rehabilitation Special Interest Group

^#^If already assessed during the Patient selection phase, the data from this previous phase can be used

*X* recommended, *O* optional/by indication, *GR* geriatric rehabilitation, *CFS* Clinical Frailty Scale, *BMI* Body Mass Index, *SNAQ65* Short Nutritional Assessment Questionnaire for 65 + , *MNA* Mini Nutritional Assessment, *MUST* Malnutrition Universal Screening Tool, *NRS* Nutritional Risk Score, *wFCI* Weighed Functional Comorbidity Index, *USER* Utrecht Scale for Evaluation of Rehabilitation, *MOCA* MOntreal Cognitive Assessment, *MMSE* Mini Mental State Examination, *Demtect* Dementia detection test, *DOS* delirium observation screening, *4AT* 4 ‘A’s test, *SQiD* single question in delirium, *HADS* Hospital Anxiety and Depression Scale, *GDS-15* Geriatric Depression Scale—15 items, *NRS-P* Numeric Rating Scale Pain, *VAS* Visual Analogue Scale, *TUG* timed up and go, *CST* chair stand test, *SPPB* short physical performance battery, *MRC* Medical Research Council scale, *PTSS* post-traumatic stress syndrome, *MWST* modified water swallowing test, *BI* Barthel Index, *FIM* Functional Independence Measure, *EQ-5D-5L* EuroQol 5-dimension-5-level, *FAI* Frenchay Activities Index, *USER-P* Utrecht Scale for Evaluation of Rehabilitation Participation, *PCR* polymerase chain reaction-test, *CSI* Caregiver Strain Index

## Conclusion

Providing tailored geriatric rehabilitation treatment to post-acute COVID-19 patients is a challenge for which the guidance presented here is designed to provide support. The present guidance for post-acute COVID-19 rehabilitation comprises two sections. The first section addresses general recommendations for geriatric rehabilitation, including general requirements for post-acute COVID-19 rehabilitation and critical aspects for quality assurance during the COVID-19 pandemic. The second section addresses specific processes and procedures, divided into (1) patient selection; (2) admission; (3) treatment; (4) discharge; and (5) follow-up and monitoring.

The present guidance has several limitations. First, although the recommendations are based on scientific evidence where available, the guidance should be treated as an expert consensus because the literature in the field is underdeveloped. Second, due to major differences among EuGMS member countries with regard to the availability and organisation of geriatric rehabilitation, combined with the heterogeneity of post-acute COVID-19 patients in geriatric rehabilitation, guidance had to be formulated in a general way, to maximise its relevance across all EuGMS member countries. Third, the authors and experts consulted for this guidance will been influenced by the experience in the country they work. However, eight countries were represented in the expert group, and experts were asked to judge the recommendations from an international view.

There is a strong need for additional evidence on COVID-19 geriatric rehabilitation including developing an understanding of risk profiles of older patients living with frailty to develop individualised treatment regimes. Furthermore, additional evidence is needed on the feasibility and effectiveness of these individualised treatment regimes. The present expert based guidance will be regularly updated based on additional evidence from practice and research.

## Supplementary Information

Below is the link to the electronic supplementary material.

Supplementary file1 (DOCX 32 KB)

## Data Availability

The manuscript is based on evidence from scientific literature combined with expert opinion.

## References

[1] Lai CC, Shih TP, Ko WC, Tang HJ, Hsueh PR (2020). Severe acute respiratory syndrome coronavirus 2 (SARS-CoV-2) and coronavirus disease-2019 (COVID-19): the epidemic and the challenges. Int J Antimicrob Agents.

[2] Liu K, Chen Y, Lin R, Han K (2020). Clinical features of COVID-19 in elderly patients: a comparison with young and middle-aged patients. J Infect.

[3] Guan W-J, Ni Z-Y, Hu Y, Liang W-H, Ou C-Q, He J-X (2020). Clinical characteristics of 2019 novel coronavirus infection in China. medRxiv.

[4] Barker-Davies RM, O’Sullivan O, Senaratne KPP, Baker P, Cranley M, Dharm-Datta S, Ellis H, Goodall D, Gough M, Lewis S, Norman J, Papadopoulou T, Roscoe D, Sherwood D, Turner P, Walker T, Mistlin A, Phillip R, Nicol AM, Bennett AN, Bahadur S (2020). The Stanford hall consensus statement for post-COVID-19 rehabilitation. Br J Sports Med.

[5] Iannaccone S, Castellazzi P, Tettamanti A, Houdayer E, Brugliera L, de Blasio F, Cimino P, Ripa M, Meloni C, Alemanno F, Scarpellini P (2020). Role of rehabilitation department for adult individuals with COVID-19: the experience of the San Raffaele Hospital of Milan. Arch Phys Med Rehabil.

[6] Pandharipande PP, Girard TD, Ely EW (2014). Long-term cognitive impairment after critical illness. N Engl J Med.

[7] Nikayin S, Rabiee A, Hashem MD, Huang M, Bienvenu OJ, Turnbull AE, Needham DM (2016). Anxiety symptoms in survivors of critical illness: a systematic review and meta-analysis. Gen Hosp Psychiatry.

[8] Parker AM, Sricharoenchai T, Raparla S, Schneck KW, Bienvenu OJ, Needham DM (2015). Posttraumatic stress disorder in critical illness survivors: a metaanalysis. Crit Care Med.

[9] Rabiee A, Nikayin S, Hashem MD, Huang M, Dinglas VD, Bienvenu OJ, Turnbull AE, Needham DM (2016). Depressive symptoms after critical illness: a systematic review and meta-analysis. Crit Care Med.

[10] Needham DM, Davidson J, Cohen H, Hopkins RO, Weinert C, Wunsch H, Zawistowski C, Bemis-Dougherty A, Berney SC, Bienvenu OJ, Brady SL, Brodsky MB, Denehy L, Elliott D, Flatley C, Harabin AL, Jones C, Louis D, Meltzer W, Muldoon SR, Palmer JB, Perme C, Robinson M, Schmidt DM, Scruth E, Spill GR, Storey CP, Render M, Votto J, Harvey MA (2012). Improving long-term outcomes after discharge from intensive care unit: report from a stakeholders' conference. Crit Care Med.

[11] Lee CM, Fan E (2012). ICU-acquired weakness: what is preventing its rehabilitation in critically ill patients?. BMC Med.

[12] Ohtake PJ, Lee AC, Scott JC, Hinman RS, Ali NA, Hinkson CR, Needham DM, Shutter L, Smith-Gabai H, Spires MC, Thiele A, Wiencek C, Smith JM (2018). Physical impairments associated with post-intensive care syndrome: systematic review based on the World Health Organization's international classification of functioning, disability and health framework. Phys Ther.

[13] Welch C, Greig C, Masud T, Wilson D, Jackson TA (2020). COVID-19 and acute sarcopenia. Aging Dis.

[14] Carfì A, Bernabei R, Landi F, Gemelli Against COVID-19 Post-Acute Care Study Group (2020). Persistent symptoms in patients after acute COVID-19. JAMA.

[15] Salini S, Russo A, Biscotti D, Broccatelli M, D’elia M, Savera G, Landi F, On behalf of Gemelli Against COVID-19 Post-Acute Care Team (2020). Professional sport players recover from coronavirus and return to competition: hopes for resuming a normal life after COVID-19 for older people. J Gerontol Geriatr.

[16] Gemelli Against COVID-19 Post-Acute Care Study Group (2020). Post-COVID-19 global health strategies: the need for an interdisciplinary approach. Aging Clin Exp Res.

[17] Lopez-Leon S, Wegman-Ostrosky T, Perelman C, Sepulveda R, Rebolledo PA, Cuapio A, Villapol S (2021). More than 50 long-term effects of COVID-19: a systematic review and meta-analysis. Sci Rep.

[18] Ceravolo MG, de Sire A, Andrenelli E, Negrini F, Negrini S (2020). Systematic rapid “living” review on rehabilitation needs due to COVID-19: update to March 31st, 2020. Eur J Phys Rehabil Med.

[19] Wade DT (2020). Rehabilitation after COVID-19: an evidence-based approach. Clin Med (Lond).

[20] Grund S, Gordon AL, van Balen R, Bachmann S, Cherubini A, Landi F, Stuck AE, Becker C, Achterberg WP, Bauer JM, Schols JMGA (2020). European consensus on core principles and future priorities for geriatric rehabilitation: consensus statement. Eur Geriatr Med.

[21] Grund S, van Wijngaarden JP, Gordon AL, Schols JMGA, Bauer JM (2020). EuGMS survey on structures of geriatric rehabilitation across Europe. Eur Geriatr Med.

[22] Bachmann S, Finger C, Huss A, Egger M, Stuck AE, Clough-Gorr KM (2010). Inpatient rehabilitation specifically designed for geriatric patients: systematic review and meta-analysis of randomised controlled trials. BMJ.

[23] Jamour M, Marburger C, Runge M, Sieber CC, Tümena T, Swoboda W (2014). Wirksamkeit geriatrischer Rehabilitation bei Hochbetagten: Eine Auswertung süddeutscher Versorgungsdaten [effectiveness of geriatric rehabilitation in the oldest old: evaluation of South German observational data]. Z Gerontol Geriatr.

[24] Freidel K, Linck-Eleftheriadis S, Röhrig B, Schilling S, Heckmann J (2017). 10 Jahre evaluation der geriatrischen rehabilitation in Rheinland-Pfalz [A 10-year evaluation of geriatric rehabilitation in Rhineland-Palatinate]. Z Gerontol Geriatr.

[25] Bordne S, Rietz C, Schulz RJ, Zank S (2020). Behavioral and emotional quality of life of patients undergoing inpatient geriatric rehabilitation. Rehabil Psychol.

[26] Gordon AL, Goodman C, Achterberg W, Barker RO, Burns E, Hanratty B, Martin FC, Meyer J, O'Neill D, Schols J, Spilsbury K (2020). Commentary: COVID in care homes-challenges and dilemmas in healthcare delivery. Age Ageing.

[27] Grund S, Gordon AL, Bauer JM, Achterberg WP, Schols JMGA (2021). The COVID rehabilitation paradox: why we need to protect and develop geriatric rehabilitation services in the face of the pandemic. Age Ageing.

[28] Grund S, Jamour M, Becker C (2021). Stationäre geriatrie: das COVID-versorgungsparadox [Inpatient geriatrics: the COVID care paradox]. Dtsch Arztebl.

[29] COVID-19 rapid guideline: managing the long-term effects of COVID-19 (2020) NICE guideline. www.nice.org.uk/guidance/ng188. Accessed 18 Dec 2020 33555768

[30] Ceravolo MG, Arienti C, de Sire A, Andrenelli E, Negrini F, Lazzarini SG, Patrini M, Negrini S, International Multiprofessional Steering Committee of Cochrane Rehabilitation REH-COVER action (2020). Rehabilitation and COVID-19: the cochrane rehabilitation 2020 rapid living systematic review. Eur J Phys Rehabil Med.

[31] Demeco A, Marotta N, Barletta M, Pino I, Marinaro C, Petraroli A, Moggio L, Ammendolia A (2020). Rehabilitation of patients post-COVID-19 infection: a literature review. J Int Med Res.

[32] Verenso (2020) Behandeladvies post-Covid-19 (geriatrische) revalidatie. Module klinische revalidatie: versie 2.1. Utrecht: Verenso; 01-12-2020. https://www.verenso.nl/_asset/_public/Thema-en-projecten/Infectieziekten/Covid-19/201201-Behandeladvies-post-COVID-19-geriatrische-revalidatie_Versie-2-1_1-december-2020.pdf

[33] Verenso (2020) Behandeladvies post-Covid-19 (geriatrische) revalidatie. Module ambulante geriatrische revalidatie: versie 1.0. Utrecht: Verenso; 01-12-2020. https://www.verenso.nl/_asset/_public/Thema-en-projecten/Infectieziekten/Covid-19/Behandeladvies-post-COVID-19_module_ambulante_GR_versie_1-0_DEFINITIEF.pdf

[34] Grund S, Caljouw MAA, Haaksma ML, Gordon AL, van Balen R, Bauer JM, Schols JMGA, Achterberg WP (2021). Pan-European study on functional and medical recovery and geriatric rehabilitation services of post-COVID-19 patients: protocol of the EU-COGER study. J Nutr Health Aging.

[35] Behandelprogramma Geriatrische COPD Revalidatie (2020) Universitair Netwerk voor de Care sector Zuid-Holland (UNC-ZH). Documenten Behandelprogramma Geriatrische COPD Revalidatie (lumc.nl)

[36] van Dam van Isselt EF (2019) Geriatric rehabilitation for older patients with COPD; integration of rehabilitation and palliative care. Doctoral thesis, Leiden University Medical Centre (LUMC), Leiden University

[37] de Graaf J, Brouwers M, Post M (2020) Behandelprogramma COVID-19 post-IC in de medisch specialistische revalidatie (De Hoogstraat). Versie 1.2, datum 18-06-2020. www.dehoogstraat.nl/wp-content/uploads/2020/06/Behandelprogramma-COVID-19-post-IC-versie-1.2-De-Hoogstraat-18-juni-2020.pdf

[38] Grund S, Gordon AL, Bauer JM, Achterberg W, Schols JMGA (2021) COVID-19 pandemic and consecutive changes in geriatric rehabilitation structures and processes—a deeper attempt to explain the COVID rehabilitation paradox. Age Ageing 10.1007/s12603-021-1716-1 PMC8691966 35067705

[39] Fewster E (2020) Care homes strategy for infection prevention and control of COVID-19 based on clear delineation of risk zones. https://bushproof.com/care-homes-strategy-for-infection-prevention-control-of-covid-19/.

[40] Rhee C, Kanjilal S, Baker M, Klompas M (2021). Duration of severe acute respiratory syndrome coronavirus 2 (SARS-CoV-2) infectivity: when is it safe to discontinue isolation?. Clin Infect Dis.

[41] Mina MJ, Peto TE, García-Fiñana M, Semple MG, Buchan IE (2021). Clarifying the evidence on SARS-CoV-2 antigen rapid tests in public health responses to COVID-19. Lancet.

[42] Scientific Advisory Group for Emergencies: Social Care Working Group (2021) Commission: What are the appropriate layers of mitigation to deploy for care homes in the context of post vaccination risk landscape? https://assets.publishing.service.gov.uk/government/uploads/system/uploads/attachment_data/file/994620/S1257_SCWG_Post_Vaccination_Mitigations.pdf

[43] Cowley A, Goldberg SE, Gordon AL, Kerr M, Logan P (2021). Exploring rehabilitation potential in older people living with frailty: a qualitative focus group study. BMC Geriatr.

[44] Hampshire A, Trender W, Chamberlain SR, Jolly AE, Grant JE, Patrick F, Mazibuko N, Williams SC, Barnby JM, Hellyer P, Mehta MA (2021). Cognitive deficits in people who have recovered from COVID-19. EClinicalMedicine.

[45] Burdick KE, Millett CE (2021). The impact of COVID-19 on cognition in severe cases highlights the need for comprehensive neuropsychological evaluations in all survivors. Neuropsychopharmacology.

[46] Rawal G, Yadav S, Kumar R (2017). Post-intensive care syndrome: an overview. J Transl Int Med.

[47] Needham DM, Dinglas VD, Morris PE, Jackson JC, Hough CL, Mendez-Tellez PA, Wozniak AW, Colantuoni E, Ely EW, Rice TW, Hopkins RO, NIH NHLBI ARDS Network (2013). Physical and cognitive performance of patients with acute lung injury 1 year after initial trophic versus full enteral feeding. EDEN trial follow-up. Am J Respir Crit Care Med.

[48] Lee M, Kang J, Jeong YJ (2020). Risk factors for post-intensive care syndrome: a systematic review and meta-analysis. Aust Crit Care.

[49] Vermeiren S, Vella-Azzopardi R, Beckwée D (2016). Frailty and the prediction of negative health outcomes: a meta-analysis. J Am Med Dir Assoc.

[50] Standing Commission on Vaccination (2021). STIKO decision on the 10th update of the COVID-19 vaccination recommendation [Ständige Impfkommission: Beschluss der STIKO zur 10. Aktualisierung der COVID-19-Impfempfehlung] Aticle in German. Epidemiol Bull.

[51] Cavanaugh AM, Spicer KB, Thoroughman D, Glick C, Winter K (2021). Reduced risk of reinfection with SARS-CoV-2 after COVID-19 vaccination—Kentucky, May-June 2021. MMWR Morb Mortal Wkly Rep.

[52] Academy of medical sciences (2021) COVID-19 : Preparing for the future. Looking ahead to winter 2021/22 and beyond, July, 2021. https://acmedsci.ac.uk/file-download/4747802

[53] Gladman JR (2008). The international classification of functioning, disability and health and its value to rehabilitation and geriatric medicine. J Chin Med Assoc.

[54] Buckle P, Micocci M, Tulloch J, Kierkegaard P, Parvulescu P, Thompson C, Spilsbury K, Allen AJ, Body R, Hayward G, Buchan I, Gordon AL (2021). COVID-19 point-of-care testing in care homes: what are the lessons for policy and practice?. Age Ageing.

