# Supplementary material for: Management of post-acute COVID-19 patients in geriatric rehabilitation: EuGMS guidance
Source: Eur Geriatr Med. 2021 Nov 20;13(1):291–304. doi: 10.1007/s41999-021-00575-4 (PMC8605452; doi:10.1007/s41999-021-00575-4)
Supplement: Supplementary file 1 — Supplementary file1 (DOCX 32 KB) [file 41999_2021_575_MOESM1_ESM.docx]

Appendix I Specific actions related to treatment goals per discipline*^#^

| *Physician*   - Monitor health risks - Monitoring medication and making the necessary adaptations - Treatment of comorbidity - Cognitive screening and (if applicable) assessing the symptoms of delirium and starting appropriate treatment - Treatment of intercurrent conditions - Informing and keeping in contact with the family/contact person/representative of the patient (if applicable) - Contact with medical specialist of general practitioner - Supporting patient and their loved ones - Medical handover at discharge - Prescribing/tapering of oxygen and (pulmonic) medication - Coordinating the multidisciplinary treatment plan - Coordinating quality assurance of the multidisciplinary team |
| --- |
| *Nurse*   - Perform intake assessment and monitor progress; - Guiding and supporting the patient and their relatives; - Informing and keeping close contact with the patients’ relatives; - Manage or decrease administration of oxygen, based on instructions of the physician; - Mobilizing the patient, based on recommendations of physiotherapist or occupational therapist; - Training in activities of daily living, based on recommendations of the occupational therapist; - Coaching patient in managing their daily energy use, based on recommendations of the occupational therapist; - Fall prevention (e.g. training, safety equipment) - Observing, checking and reporting on general health status of the patient; - Coaching independent practice therapy - Ensure clear handover/communication to other care professionals |
| *Physiotherapist*   - Perform intake assessment and monitor progress; - Increasing capacity by doing respiratory muscle training - Training of coughing and breathing techniques (only in case of respiratory tract obstruction caused by sputum/muscus) - Mobilising - Training the musculoskeletal system - Fall prevention (e.g. training, safety equipment) - Relaxation exercises - Ensure clear handover/communication to other care professionals |
| *Occupational therapist*   - Perform intake assessment and monitor progress; - ADL-training - Assessment of home environment - Prescribing and training the use of (medical) aids - Energy management - Cognitive screening - Fall prevention (e.g. training, safety equipment) - Ensure clear handover/communication to other care professionals |
| *Dietician*   - Perform intake assessment and monitor progress; - Healthy nutritional status and optimize protein- and energy food intake - Ensure clear handover/communication to other care professionals |
| *Speech therapist*   - Perform intake assessment and monitor progress; - Practice safe swallowing - Breathing exercises - Attention for voice problems after intubation - Breath control and voice - Ensure clear handover/communication to other care professionals |
| *Psychologist*   - Perform intake assessment, and monitor progress; - Gaining insight in cognitive changes and learning strategies to compensate for this. - Assessing the mood of the patient (timely diagnosis and treatment of depression, fear and PTSS) - assessing possible impairing and promoting psychological factors (coping) - Support in processing experiences around suffering and fear of dying - Ensure clear handover/communication to other care professionals |
| *Social worker*   - Perform intake assessment. and monitor progress; - Assessing the burden of the family/informal caregivers (if applicable) - Psychological status family/ informal caregivers (if applicable) - Supporting patient and family (if applicable) with social problems, such as financial problems due to COVID-19 - Support in processing experiences around suffering and fear of dying - Mapping sources for resilience and supporting resilience - Supporting to overcome COVID-19 as life event - Exploring questions regarding identity, values and world view - Ensure clear handover/communication to other care professionals |

* Based on Verenso (2020) and supplemented and adapted by the EuGMS Geriatric Rehabilitation Special Interest Group.

# The disciplines involved in the treatment of COVID-19 patients may differ per patient based on the specific symptoms and comorbidities of the patient. For care facilities with fewer disciplines in the multidisciplinary teams, certain goals may be assigned to other disciplines if possible and feasible. In addition, care professionals can collaborate with disciplines from other care organisations. Within geriatric rehabilitation the interchange between disciplines is very important and the care professionals involved in geriatric rehabilitation should have a flexible and interdisciplinary work approach.
